# Treatment of Cholera

**Published:** 1849-09

**Authors:** C. A. Finley

**Affiliations:** Surgeon, U. S. A.; Newport Barracks, Ky.


*Treatment of Cholera.* By C. A. Finley, M. D., Surgeon, U. S. A.

To the Editors of the Medical Examiner:

Gentlemen,—Having done me the honor to publish in your journal my report of the treatment of two cases of Asiatic cholera, together with my view of the inapplicability of opium to a well marked, or fully developed case of that disease—permit me to lay before you, in the following extract from my quarterly sick report, the results of my treatment.

"It will be seen in this report, that there have been sixty-six cases of Asiatic cholera under treatment; these were—even the mildest—characterized by the discharges peculiar to that disease. The doubtful cases of diarrhœa have been placed under the head of diarrhœa. The treatment, in every case which has occurred since my special report, has been that laid down in that report—the free exhibition of calomel and camphor, and the quinine after reaction was fairly established. Four of the sixty-six cases were lost; among the recoveries were many in incipient collapse, several in whom the pulsation of the radial artery could not be discerned when the patients were admitted. Ptyalism occurred in ten or twelve cases; in only three cases was it severe, or of more than ten days' continuance. As soon as the dejections gave evidence of the restoration of the biliary secretion, the ol. ricini was given freely, and assisted by enemata, whilst every four or five hours quinine and camphor in doses of five grains each were administered. Under this treatment few cases remained in the Hospital longer than five days."

There were seven cases of the sixty-six, in which calomel to the amount of three hundred grains was given, and the period of their continuance in the Hospital was as follows: Rhette thirteen days, Stark three, Douthett twenty, Brown three, Hoeffer nine, Kippe five, Doran thirteen.

*Newport Barracks, Ky., August 15th,* 1849.

